# Formulation and Evaluation of Rizatriptan Benzoate Mouth Disintegrating Tablets

**DOI:** 10.4103/0250-474X.62253

**Published:** 2010

**Authors:** R. V. Keny, Chrisma Desouza, C. F. Lourenco

**Affiliations:** Department of Pharmaceutics, Goa College of Pharmacy, 18^th^ June Road, Panaji, Goa-403 001, India

**Keywords:** Direct compression, *in vitro* and *in vivo* disintegration time, mouth disintegrating, rizatriptan benzoate, wetting time

## Abstract

The present investigation deals with development of mouth disintegrating tablets of rizatriptan benzoate to produce the intended benefits. Mouth disintegrating tablets of rizatriptan benzoate were prepared using superdisintegrants crospovidone, carboxymethylcellulose calcium, Indion 414 and Indion 234 using the direct compression method. The tablets prepared were evaluated for thickness, uniformity of weight, content uniformity, hardness, friability, wetting time, *in vitro* and *in vivo* disintegration time, mouth feel, *in vitro* drug release and assay by high performance liquid chromatography. The tablets disintegrated *in vitro* and *in vivo* within 4 to 7 s and 6 to 19 s, respectively. Almost 90% of drug was released from all formulations within 20 min. The drug release from the formulations followed first order kinetics. Stability studies of the tablets at 40±2°/75%±5% RH for 1 mo showed non significant drug loss. The formulation containing combination of crospovidone and Indion 234 was found to give the best results. Apart from fulfilling all official and other specifications, the tablets exhibited higher rate of release.

The new generation anti-migraine drug, rizatriptan benzoate is a potent and selective 5-hydroxytryptamine_1B/1D_ receptor agonist and is considered more effective than the traditional triptans for the treatment of acute migraine attack[1]. Chemically it is 3-[2-(dimethylamino) ethyl]-5-(1H-1,2,4-triazol-1-ylmethyl)indole monobenzoate. A 10 mg dose of rizatriptan benzoate is equipotent to a 100 mg of sumatriptan, the traditional antimigraine drug[2]. The bioavailability of rizatriptan benzoate is about 45% which is superior to a poor 14-17% of sumatriptan. It has a very fast onset of action within one hour of intake, providing immediate relief from migraine[1]. As migraine sufferers have markedly reduced functional ability, they would be benefited from acute treatment that helps them to resume their functional activities as quickly as possible.

Recent developments in technology have presented viable dosage alternatives for paediatric, geriatric, bedridden, nauseous or non compliant patients[3], who face difficulty in swallowing or chewing solid dosage forms and are unwilling to take solid preparations due to a fear of choking[4]. Hence, mouth dissolving/disintegrating tablets are a perfect fit for them. Superdisintegrants added in the formulation increase the drug release, thus increasing the bioavailability of drug[5]. Mouth disintegrating tablets when placed in the mouth, disintegrate instantaneously, releasing the drug, which dissolves or disperses in the saliva and can be swallowed as a liquid, without the aid of water[3,6,7]. Also, this dosage form offers an advantage of convenience of administration while travelling where there may not be an access to water[3]. Moreover, this dosage form combines the advantages of both liquid and tablet formulation. Some drugs are absorbed from the mouth, pharynx and oesophagus as the saliva passes down into the stomach. In such cases, bioavailability of drug is significantly greater than that observed from conventional tablet dosage form. This system of drug delivery allows children, elderly, and the general population to take their medications discretely wherever and whenever needed, much eliminating the factor of patient non-compliance. The benefits, in terms of patient compliance, rapid onset of action, increased bioavailability, and good stability make these tablets popular as a dosage form of choice in the current market[8,9].

## MATERIALS AND METHODS

Rizatriptan Benzoate was obtained as a gift sample from Cipla Ltd. Kurkumbh, Pune. Indion 234 was a sample from Wyeth Ltd., Verna, Goa. Indion 414 was gifted by Ion exchange (India) Ltd, Mumbai. Carboxymethylcellulose calcium was provided by Arihant Trading Co., Mumbai. Glenmark Pharmaceuticals Ltd., Colvale, Goa gifted crospovidone, avicel PH-102, mannitol, magnesium stearate, talc and aspartame. All other chemicals used were of analytical grade.

### Preformulations:

Identification of the drug was carried out by FTIR (Shimadzu 8101A, Mumbai). Standardization of the drug was carried out using UV/Vis spectrophotometer (Perkin Elmer, Mumbai). IR spectral analysis of the formulations was performed to assess drug excipient compatibility. Preliminary studies were carried out on the tablets using different concentrations of superdisintegrants. Thus, after evaluation of the quality parameters and subjecting to *in vitro* disintegration and *in vitro* dissolution studies the final concentrations of the superdisintegrants were optimized. Based on this preformulation data the optimized formulations for further investigations were decided.

### Formulation:

Mouth disintegrating tablets each containing rizatriptan benzoate equivalent to 5 mg of rizatriptan, were prepared using superdisintegrants crospovidone, carboxymethylcellulose calcium, Indion 414, Indion 234 and their combinations, by direct compression technique[10] according to the formulae given in Table 1. The average weight of the tablet was found to be 150 mg. The ingredients were sifted through a 40# mesh and then the required quantities weighed. All the ingredients except magnesium stearate and the flavoring agents were uniformly blended. After mixing the drug and the excipients for 20 min, magnesium stearate and the flavoring agents were added and further mixed for additional 2 min. The tablet mixture was then compressed (8 mm diameter, concave punches) using a single punch tablet compression machine (Cadmach, Ahmedabad). A batch of 100 tablets was prepared for each of the formulations.

**TABLE 1:** COMPOSITION OF DIFFERENT BATCHES OF MOUTH DISINTEGRATING TABLETS OF RIZATRIPTAN BENZOATE

Ingredients	Quantity (mg) present in each tablet									
	
	F1	F2	F3	F4	F5	F6	F7	F8	F9	F10
Rizatriptan Benzoate	7.265	7.265	7.265	7.265	7.265	7.265	7.265	7.265	7.265	7.265
Crospovidone	6	-	-	3	-	3	-	3	-	-
CMC calcium	-	6	-	3	3	-	-	-	3	-
Indion 414	-	-	3	-	3	3	-	-	-	3
Indion 234	-	-	-	-	-	-	3	3	3	3
Avicel PH102	112	112	115	112	112	112	115	112	112	112
Mannitol	15	15	15	15	15	15	15	15	15	15
Magnesium stearate	0.75	0.75	0.75	0.75	0.75	0.75	0.75	0.75	0.75	0.75
Talc	1.5	1.5	1.5	1.5	1.5	1.5	1.5	1.5	1.5	1.5
Aspartame	3	3	3	3	3	3	3	3	3	3
Mixed fruit dry-mix powder	3	3	3	3	3	3	3	3	3	3
American mint DC 213	1.5	1.5	1.5	1.5	1.5	1.5	1.5	1.5	1.5	1.5

Defined bulk weight per tablet is 150 mg containing rizatriptan benzoate equivalent to 5 mg of rizatriptan. F1 to F10 represents the various tablet formulations of rizatriptan benzoate.

### Evaluation:

The prepared tablets were evaluated for thickness and weight variation as per compendial procedure. The thickness was determined for 10 tablets with the help of a digital Vernier caliper (Mututoyo, Japan). For the weight variation, twenty tablets were selected at random and assessed individually using an electronic balance (Precisa 310M, Switzerland). The individual weights were compared with the average weight for determination of weight variation[11]. The fracture strength, which is defined as the force, required to break a tablet by radial compression, was determined for 10 tablets using Monsanto hardness tester (Campbell Electronics, Mumbai)[12]. Friability of 20 tablets was measured using a Roche Friabilator (Electrolab, Mumbai)[11]. Twenty preweighed tablets were rotated at 25 rpm for 4 min. The tablets were then reweighed after removal of fines and the percentage of weight loss was calculated. Wetting time was measured by placing a tablet on a piece of tissue paper folded twice in a small petridish (i.d.= 6.5 cm) containing 5 ml of phosphate buffer pH 6.8, and the time required for complete wetting was then measured[13]. *In vitro* disintegration time[9] was determined by dropping a tablet in a beaker containing 5 ml of pH 6.8 phosphate buffer at 37±0.5° and the time required for complete dispersion was determined. The *in vivo* disintegration time was measured in five healthy volunteers. The end point for the disintegration in the mouth is the time when the tablet placed on the tongue disintegrates until no lumps remain. While testing, the volunteers kept the tablets motionless on their tongues[14]. IR spectra of pure drug rizatriptan benzoate and its formulations were obtained by potassium bromide pellet method using FTIR spectrophotometer in order to rule out drug carrier interactions.

The assay of tablets was performed by HPLC using Spectra System SCM1000 High Performance Liquid Chromatograph and Cosmosil C8 packed column (250×4.6 mm, 5 μm). The mobile phase used was buffer:acetonitrile (85:15)[15]. The buffer comprised of a mixture of 0.05 M NaH_2_ PO_4_ monohydrate and 0.05 M sodium perchlorate, adjusted to pH 3.55±0.10 with perchloric acid. An aliquot from powdered tablets each containing 5 mg of rizatriptan benzoate was used. The flow rate was set as 1.5 ml/min with a column temperature of 25° and a run time of 22 min. The injected volume was 20 μl and an UV detector was used (225 nm).

The standard calibration curve graph was obtained by preparing aliquots of standard solution of rizatriptan benzoate in 0.1 N HCl (pH 1.2) and the absorbance at 280 nm was measured after suitable dilution using UV/Vis spectrophotometer. For content uniformity test, ten tablets of each batch were weighed and powdered. Aliquot of this powder containing rizatriptan benzoate equivalent to 5 mg of rizatriptan was accurately weighed, suspended in approximately 50 ml of 0.1 N HCl and shaken for 15 min. Final volume was adjusted to 100 ml with 0.1 N HCl and filtered (Whatmann No.1 filter paper). From this 10 ml was diluted to 100 ml. The final volume was made by taking 2 ml of above solution and diluted to 10 ml with 0.1 N HCl. Absorbance of this solution was recorded at 280 nm using UV/Vis spectrophotometer against a reagent blank and the content was compared from a calibration curve prepared with standard rizatriptan benzoate in the same medium. The mean percent drug content was calculated as an average of three determinations[16].

### *In vitro* release rate studies:

**Fig. 1:**
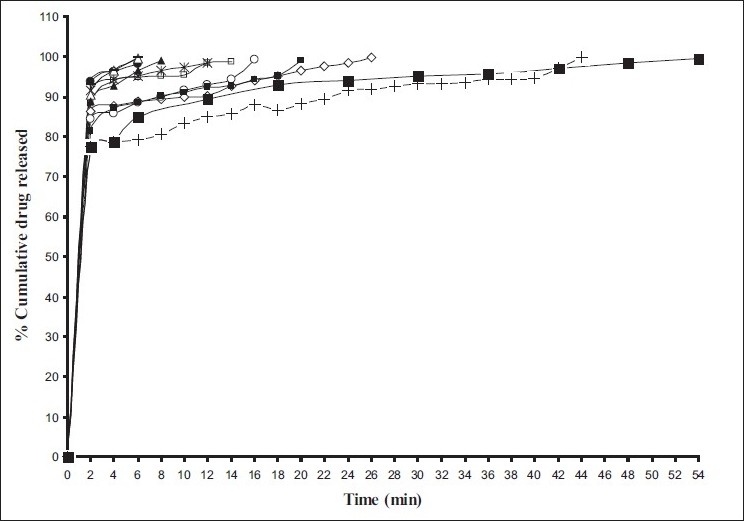
*In vitro* cumulative percent release profiles of rizatriptan benzoate Prepared formulations, F1 (-•-), F2 (-◊-), F3 (-*-), F4 (-¤-), F5 (-Δ-), F6 (-■-), F7 (-+-), F8 (-▬-), F9 (-▲-), F10 (-□-) and marketed conventional tablet (-■-).

*In vitro* dissolution of rizatriptan benzoate mouth disintegrating tablets was studied in USP dissolution test apparatus Type II (Labindia, Mumbai) employing a paddle stirrer at 50 rpm using 500 ml of 0.1 N HCl (pH 1.2) at 37±0.5° as dissolution medium[17]. One tablet was used in each test. Aliquots of dissolution medium (5 ml) were withdrawn at specific intervals of time (2 min) and analyzed for drug content by measuring the absorbance at 280 nm. The volume withdrawn at each time interval was replaced with fresh quantity of dissolution medium. Cumulative percent drug released was calculated and plotted against time. Dissolution of all the designed formulations and the conventional marketed formulation were evaluated[18]. The results are shown in fig. 1.

### Stability studies:

Stability study of the prepared tablets was performed at 40±2°/75%±5% RH for a period of 1 mo. The tablets were then evaluated for hardness, friability, *in vitro* disintegration time, wetting time, uniformity of content and assay[19].

## RESULTS AND DISCUSSION

Mouth disintegrating tablets each containing rizatriptan benzoate equivalent to 5 mg of rizatriptan, were prepared by direct compression method employing crospovidone, carboxymethylcellulose calcium, Indion 414 and Indion 234 as superdisintegrants in different ratios, either alone or in combination. Directly compressible excipients, avicel pH102 and mannitol were used as diluents to enhance mouth feel. A total of ten formulations were designed and evaluated in addition to the marketed preparation (Table 1).

**Fig. 2:**
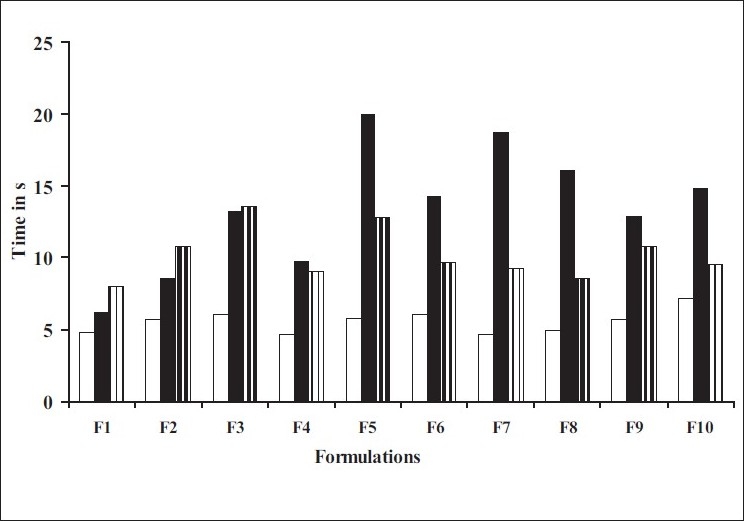
Comparison between *in vitro* and *in vivo* disintegration time and wetting time of the prepared formulations *In vitro* disintegration time (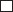), *in vivo* disintegration time (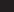) and wetting time (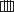).

Table 2 depicts all the tablet parameters evaluated. The thickness of the prepared tablets was found to be in the range of 3.61±0.054 to 3.68±0.087 mm, while the weight of all the tablets was found to be in the range of 149.0±1.395 to 150.9±1.373 mg. Hardness of the tablets was found to be in the range of 2.05±0.11 to 2.25±0.25 kg/cm^2^ and percentage weight loss in the friability test was less than 1% in all the batches, which was an indication of good mechanical resistance of the tablets. Wetting time which is an important criteria for understanding the capacity disintegrants to swell in the presence of little amount of water was found to be in the range of 8.04±1.823 to 13.60±0.118 s (fig. 2). All the tablet formulations disintegrated rapidly *in vitro* within 4.65±0.113 to 7.16±0.798 s and *in vivo* within 6.27±0.071 to 19.94±1.961 s as shown in fig. 2. The tablets containing individual superdisintegrants showed *in vitro* disintegration time in the following order, Indion234>Crospovidone>carboxymethylcellulose calcium>Indion 414. Nevertheless, all four proved to be very effective disintegrants. The tablets containing crospovidone and calcium CMC in combination, showed faster disintegration than tablets containing crospovidone alone, calcium CMC alone and their combinations with other superdisintegrants. Indion 414 showed comparable disintegration when used alone and in combination with Crospovidone, but fastest disintegration was seen in its combination with calcium CMC than with others. Indion 234 also showed satisfactory disintegration time when used alone (18-19 s) and in combination with Crospovidone (15-16 s), compared to its combination with other superdisintegrants. IR spectroscopic studies indicated that the drug is compatible with all the excipients. The IR spectrum of the formulations showed all the characteristic peaks of rizatriptan pure drug, thus confirming that no interaction of drug occurred with the components of the formulation.

**TABLE 2:** EVALUATION OF TABLET PARAMETERS

Formulations	Thickness* (mm) ± SD	Hardness* (Kg/cm^2^) ± SD	Friability# (%) ± SD	Weight Variation# (mg) ± SD	Rizatriptan content§ (mg)	Assay	

						Rizatriptan content§ (%) ± SD	Rizatriptan content§ mg/tab
F1	3.66±0.03	2.20±0.27	0.132	150.8±1.08	4.989	100.99±0.70	5.049
F2	3.63±0.06	2.10±0.22	0.134	150.9±1.37	4.939	101.33±0.79	5.067
F3	3.63±0.02	2.05±0.11	0.133	150.6±0.83	4.897	98.85±1.0	4.943
F4	3.64±0.02	2.10±0.14	0.200	149.4±1.43	4.915	98.51±0.78	4.926
F5	3.64±0.05	2.20±0.21	0.095	150.3±1.02	4.904	98.06±0.59	4.903
F6	3.68±0.09	2.25±0.25	0.067	149.9±1.37	4.913	98.27±0.74	4.912
F7	3.65±0.06	2.20±0.27	0.066	149.0±1.40	4.924	97.72±0.56	4.886
F8	3.63±0.04	2.30±0.21	0.131	150.8±0.41	4.951	98.99±0.64	4.949
F9	3.58±0.04	2.25±0.18	0.066	150.5±0.83	4.888	98.19±0.83	4.909
F10	3.61±0.05	2.05±0.11	0.066	150.4±0.81	4.901	99.02±0.75	4.951

F1 to F10 represents the various tablet formulations of rizatriptan benzoate.

* Each value represents a mean of 10 determinations.

# Each value represents a mean of 20 determinations.

§ Each value represents a mean of 3 determinations

As seen from Table 2, the assay revealed the tablets to contain rizatriptan benzoate equivalent to rizatriptan between 97.72±0.56 to 101.33±0.79% of the labeled claim. Solutions of rizatriptan benzoate obeyed Beer's law in the concentration range of 5-50 μg/ml. All the tablets subjected to uniformity of drug content, were found to contain rizatriptan benzoate equivalent to rizatriptan within 100±5% of the labeled claim (Table 2).

Rizatriptan Benzoate release was significantly faster from all the prepared formulations as compared to marketed conventional tablet formulation (fig. 1). Release rate of rizatriptan benzoate from the formulations F1, F5, F8, F9 was found to be faster than F2, F3, F4, F6, F7, F10 respectively, and in turn conventional marketed formulation as shown in fig. 1 and Table 3.

**TABLE 3:** *IN VITRO* RELEASE PROFILES OF RIZATRIPTAN BENZOATE FROM PREPARED FORMULATIONS AND MARKETED CONVENTIONAL TABLET

Time (min)	Cumulative % drug releasedx										
	
	F1	F2	F3	F4	F5	F6	F7	F8	F9	F10	Market Product
2	93.84	86.43	91.56	84.44	90.61	81.49	77.51	92.84	88.76	88.19	77.51
4	96.26	87.81	94.26	85.67	96.64	87.14	78.69	96.54	92.65	94.12	78.74
6	98.20	88.76	95.02	88.66	99.49	88.57	79.22	99.82	96.54	94.79	84.87
8	-	89.33	96.39	89.94	-	90.28	80.45	-	98.87	95.17	-
10	-	89.80	97.44	91.70	-	90.94	83.35	-	-	95.55	-
12	-	90.09	98.54	92.93	-	92.46	84.91	-	-	98.44	89.47
14	-	92.69	-	94.41	-	92.60	85.82	-	-	98.77−	-
16	-	94.03	-	99.25	-	94.22	88.05	-	-	-	-
18	-	95.02	-	-	-	95.21	86.62	-	-	-	92.93
20	-	96.49	-	-	-	98.87	88.23	-	-	-	-
22	-	97.49	-	-	-	-	89.28	-	-	-	-
24	-	98.53	-	-	-	-	91.51	-	-	-	93.93
26	-	99.86	-	-	-	-	91.84	-	-	-	-
28	-	-	-	-	-	-	92.65	-	-	-	-
30	-	-	-	-	-	-	93.12	-	-	-	95.26
32	-	-	-	-	-	-	93.27	-	-	-	-
34	-	-	-	-	-	-	93.55	-	-	-	-
36	-	-	-	-	-	-	94.31	-	-	-	95.59
38	-	-	-	-	-	-	94.41	-	-	-	-
40	-	-	-	-	-	-	94.55	-	-	-	-
42	-	-	-	-	-	-	96.97	-	-	-	97.02
44	-	-	-	-	-	-	99.72	-	-	-	-
46	-	-	-	-	-	-	-	-	-	-	-
48	-	-	-	-	-	-	-	-	-	-	98.49
50	-	-	-	-	-	-	-	-	-	-	-
52	-	-	-	-	-	-	-	-	-	-	-
54	-	-	-	-	-	-	-	-	-	-	99.44
56	-	-	-	-	-	-	-	-	-	-	-

F1 to F10 represents the various tablet formulations of rizatriptan benzoate and market product is the conventional commercial tablet formulation of rizatriptan benzoate.

In order to describe the kinetics of the release process of drug in all the formulations, equations such as zero-order and first-order rate equations were used. Zero-order rate equation describes the systems where the release rate is independent of the concentrations of the dissolved species[20], while the first-order equation describes the release from systems where dissolution rate is dependent on the concentration of the dissolving species[21]. It is evident from Table 4, that the drug release process is not zero-order in nature. This indicates that the dissolution rate of the drug is not independent of the amount of drug available for dissolution and diffusion from the matrix. The dissolution data of all formulations when fitted in accordance with the first-order equation it is evident that a linear relationship was obtained with ‘r’ (correlation coefficient) value close to unity and higher than ‘r’ obtained from the zero-order equation for all formulations (Table 4), showing that the release is an apparent first-order process. This indicates that the amount of drug released is dependent on the matrix drug load[22].

**TABLE 4:** KINETIC VALUES OBTAINED FROM ZERO-ORDER AND FIRST-ORDER PLOTS OF FORMULATIONS, F1 TO F10

Formulation Code	Zero order plots r^a^	First order plots r^a^
F1	0.636	0.988
F2	0.349	0.886
F3	0.331	0.971
F4	0.439	0.963
F5	0.672	1.000
F6	0.395	0.772
F7	0.386	0.969
F8	0.657	1.000
F9	0.590	0.961
F10	0.417	0.885

F1 to F10 represents the various tablet formulations of rizatriptan benzoate.

a Correlation coefficient.

Formulation F4 containing crospovidone and calcium CMC in combination exhibited fastest disintegration time of 4.65±0.11 s as shown in fig. 2. However, the drug release at 6 min was only 88.66% as compared to 99.82% within the same time from F8 containing crospovidone and Indion 234 in combination while exhibiting a comparable disintegration time of 4.94±0.36 s. Formulation F8 proved to be the best devised formulation.

Indion 234 when used alone in formulation F7 showed good disintegration time of 4.66±0.148 s, but extended the duration of complete drug release to 44 min. This could be due to complexation of Indion 234 with rizatriptan benzoate, as Indion 234 is also a taste masking agent used for taste masking of drugs having an objectionable taste by forming a complex with the drug. However, Indion 234 in formulation F8 in combination with crospovidone showed a good disintegration time of 4.94±0.33s and complete drug release was achieved in 6 min. This may be attributed to elimination or blockade of complexing activity of Indion 234 with the drug, due to the presence of crospovidone, resulting in an excellent dissolution profile with synergistic activity of superdisintegrant.

In stability indicating HPLC assay, the 1 mo tablet samples showed a rizatriptan content of 97.42±1.02% to 100.15±0.42% of the labeled claim. Also, there was absence of any extra peak in the chromatogram which proved that formulations were stable at the end of 1 month period. The drug content by UV/Vis spectrophotometry was found to be between 97.06±0.28 to 97.94±0.36% of the labeled claim. Also the other physicochemical properties were found to be within the limits. Thus, the short-term stability studies of the prepared formulations indicated that there are no significant changes in drug content and other parameters evaluated at the end of 1 mo period (*P*<0.005).

In conclusion, a stable, effective and pleasant tasting mouth disintegrating tablet, exhibiting an excellent disintegration time and dissolution profile, was formulated using a combination of crospovidone (4%) and Indion 234 (2%) as superdisintegrants. Even though the excipients employed are well known and established, they have not been used with rizatriptan benzoate for formulating mouth disintegrating tablets. Also such formulation is not available in the market. The formulation was developed with the aim of providing patients suffering from migraine, a convenient means of taking their medication, whenever and wherever needed, for immediate relief of migraine. The main motive was to achieve fast disintegration and immediate drug release, using an economic, industry feasible method involving conventional tabletting equipments. Undoubtedly, all the formulations showed good disintegration time, apart from fulfilling all compendial and other standard specifications, and exhibited higher release rates of rizatriptan benzoate.
